# Network of Soil Fungi and the Microfauna Community under Diverse Anthropic Disturbances under *Chrysopogon zizanioides* Planting in the Reservoir

**DOI:** 10.3390/plants13030393

**Published:** 2024-01-29

**Authors:** Xiaoyue Lin, Xuemei Han, Jiading Yang, Fengyu Liu, Yuying Li, Zhaojin Chen

**Affiliations:** 1 Ministry of Education Key Laboratory for Ecology of Tropical Islands, Key Laboratory of Tropical Animal and Plant Ecology of Hainan Province, College of Life Sciences, Hainan Normal University, Haikou 571158, China; linxiaoyue9201@163.com (X.L.); yangjiaidng1026@163.com (J.Y.); 13735028967@163.com (F.L.); 2 College of Life Science and Agricultural Engineering, Nanyang Normal University, Nanyang 473061, China; 3 School of Water Resources and Environment Engineering, Nanyang Normal University, Nanyang 473061, China; lyying200508@163.com (Y.L.); zhaojin_chen@163.com (Z.C.)

**Keywords:** Danjiangkou reservoir, soil eukaryotic microbes, 18S rDNA, hydro-fluctuation belt, co-occurrence network

## Abstract

The reservoir coastal zone is the transitional zone between the terrestrial ecosystem and the aquatic ecosystem. Soil is an essential part of the terrestrial ecosystem and vital for life on Earth. To understand the composition and diversity of the soil eukaryotic microbial community under the background of artificial planting of *Chrysopogon zizanioides* in various habitats after reservoir construction, including the original habitat (OH), the hydro-fluctuation belt (HB), and the road slope (RS), and to analyze the interaction between the main groups of eukaryotic microorganisms, this study conducted 18S rDNA amplification high-throughput sequencing of the soil eukaryotic microbial community. The study found that the dominant phylum of eukaryotic microorganisms in the three habitats was consistent, but there were significant differences in the community and diversity of eukaryotic microorganisms in the three habitats. The differences in fungal communities between sample sites were greater than those of soil microfauna. Correlation analysis showed that nitrogen, phosphorus, and organic matter were significantly correlated with eukaryotic microbial diversity, with alkaline-hydrolyzed nitrogen and total phosphorus significantly correlated with fungal communities and pH and water content correlated with soil microfauna. Co-occurrence network analysis found that the interactions between fungi and the correlation between fungi and soil microfauna dominated the eukaryotic microbial community, and the interactions between eukaryotic microbes in different habitats were dominated by positive correlations. After the construction of the reservoir, the newly formed hydro-fluctuation belt reduced the types of interrelationships between fungi and microfauna compared to the original habitat. The road slope provided protection of the supporting project for the reservoir construction, although there was also planted vegetation. Eukaryotic microbes declined significantly due to the damage to and loss of the organic layer, and the decline in microfauna was the most significant, resulting in a simple structure of the soil food web, which affects the function and stability of the soil ecosystem.

## 1. Introduction

Soil is a key component of the terrestrial ecosystem and crucial for life on Earth [1], as well as the foundation of environmental health and food security [2]. Similarly, eukaryotic microorganisms make up the majority of natural microbes, and their close relationship to the sustainability of soil-based ecosystems and biogeochemical processes is an important position in soil biomes [3,4]. Eukaryotic microorganisms in soil mainly include fungi, protists, metazoans, and algae, and different eukaryotic microorganisms change soil nutrient cycling by secreting different extracellular enzymes, which in turn affect soil function and stability and play a key role in the material cycle and energy flow of the ecosystem [5,6,7]. Fungi constitute most of the biomass of soil eukaryotic microorganisms and play a vital role in the formation and decomposition of soil organic matter, the circulation and utilization of nutrients, the maintenance and improvement of soil fertility, and the improvement and restoration of the ecological environment and also occupy an extremely important position in maintaining the stability of soil ecosystems and biodiversity [8]. In the material cycle of soil ecosystems, soil organisms are essential consumers. They improve the physical structure of the soil, aid in the cycling of nutrients and the stability of organic carbon, and contribute to the overall health of crops [9,10].

Soil eukaryotic microorganisms can detect soil changes under natural or anthropogenic interference, and variables related to the environment (such as temperature, moisture, organic matter and texture) and abiotic factors (such as anthropogenic activities) have substantial effects on their population dynamics [11,12,13]. A range of variables in the environment affect the structure of fungal communities, and there is often interaction between the effects of soil physicochemical qualities and fungal community structure [14]. Land use type can affect the soil structure and physicochemical characteristics by changing soil water, fertilizer, gas, heat, and other conditions, causing changes in soil microbial community structure and function, thereby affecting the stability of ecosystems. When studying soil microorganisms in temperate forests, Wallenstein et al. [15] believed that the community composition of fungi is highly sensitive to the increase in nitrogen. In a previous study, it was reported that a relationship exists between soil fungi and physicochemical factors in different seasons, and soil organic matter, total nitrogen, available nitrogen, available phosphorus, available potassium, and moisture content were closely related to changes in fungal community structure [16]. Similarly, characteristics of the soil substrate mostly shaped the diversity of the fungal community, while water emerged as the key element influencing the shift in fungal community structure after wetland restoration [17]. The number of individuals and groups of soil microfauna communities is influenced by human activities in their respective ecosystems. Long-term interference from human factors can lead to changes in soil pH, water content, organic matter, and porosity. When the natural environment changes or is strongly disturbed by humans, the community structure of soil microfauna usually changes. Organic matter is the main environmental factor driving changes in soil microfauna communities when studying agroforestry systems [18]. Liu et al. [19] pointed out that when the intensity of human disturbance is high, the diversity of soil microfauna in farmland will decrease. In the Nanniwan Wetland on the Loess Plateau, Luo et al. [20] examined the relationship between soil organisms and six physicochemical factors, including pH levels, soil organic matter, and total nitrogen. Results indicated that the distribution of soil organisms was significantly influenced by the pH and water content of the soil [20].

Soil eukaryotic microorganisms do not exist in isolation. Species can exhibit complex coexistence patterns through various interactions, such as mutualism, parasitism, competition, and predation, forming a complex interaction network system, which is crucial for the ecological service function of soil [21,22]. Soil microfauna are the main consumers of fungi in the soil, and there is a potential predatory relationship between the two. The abundance, phylogenetic diversity, and community composition of soil microfauna are significantly correlated with the biological characteristics of fungal taxonomic units and are important regulators of fungal communities [23]. A continental-scale network analysis showed that the interactions between fungi and soil microfauna can alter the cycle of nutrients in terrestrial ecosystems [24]. Similarly, another comparable study showed that the use of organic fertilizers increased the complexity of ecological interactions between soil microfauna and microorganisms. Their study of how human activity affects farming soil ecosystems revealed a more intricate web of relationships between important decomposers (fungi and bacteria) and predators (microorganisms and protozoa) [25]. Network analysis is an effective tool for elucidating potential interactions between organisms, and co-occurrence network analysis is a powerful bioinformatics analysis technique that has been widely applied to explore the complex interactions between microbial communities and the organization and dynamics of biota in soil, subsidence zones, sediments, and water environments [26,27]. The study of ecological modules based on soil microbial co-occurrence network analysis is an effective strategy for exploring the multifunctional relationship between soil microbial communities and ecosystems [28]. Using co-occurrence network analysis to elucidate the interaction relationship between soil eukaryotic microbial communities will also be of great significance for maintaining soil productivity and sustainability.

The Danjiangkou Reservoir is the source of water for the world’s largest water transfer project—the Middle Route of China’s South to North Water Diversion Project. On 16 October 2014, the water level of the Danjiangkou Reservoir increased significantly, and the original water level zone was submerged, forming a new hydro-fluctuation belt at higher altitudes. The new hydro-fluctuation belt has become the most fragile new zone in the reservoir ecosystem [29]. In the meantime, the restoration and development of several embankment slopes have resulted in an unavoidable impact on the initial natural habitat due to reservoir construction. A previous study showed the spatiotemporal changes in habitat quality in the upper Miyun Reservoir watershed between 2005 and 2015 and found that overall habitat conditions have improved over the last ten years. It is noteworthy, meanwhile, that some regions in the watershed’s southeast have seen notable habitat deterioration [30]. In addition, New et al. [31] found that the Three Gorges Dam will have significant negative impacts on the reservoir and surrounding ecosystems, as well as the environment of the entire Yangtze River basin, throughout its entire life cycle and for several years after the dam is shut down. Vetiver grass is drought resistant, waterlogging resistant, barren resistant, and salt alkali resistant. Its lush network root structure gives it good soil and water retention ability [32,33]. It is widely used in road and riverbank slope protection and other ecological restoration projects and has been listed as a designated grass species for soil and water conservation in the Danjiangkou Reservoir area and upstream. In order to understand the changes in the soil food web structure and function of newly formed habitats after reservoir construction under the background of vetiver grass cultivation, we analyzed the differences in soil chemical characteristics and eukaryotic microbial community composition under different vetiver grass planting habitats and used co-occurrence networks to explore the interaction patterns between soil microfauna and fungi, as well as different groups of eukaryotic microorganisms. This has important reference significance for evaluating the impact of reservoir construction on the diversity of eukaryotic microorganisms, maintaining the stability and service functions of the regional ecosystem, and assessing the restoration and reconstruction of ecosystem functions after reservoir construction.

## 2. Materials and Methods

### 2.1. Overview of the Research Area and Sample Setting

The research site is located in Shiqiao Village (32°50′ N and 111°35′ E), Madeng Township, Xichuan County, Henan Province, near the Danjiangkou Reservoir. It belongs to a monsoon climate that transitions from the northern subtropical zone to the temperate zone with a mild climate, four distinct seasons, abundant rainfall, an average annual sunshine time of 2046 h, precipitation of about 804 mm, temperature of 15.8 °C, and a frost-free period of 228 days. The soil is mainly composed of yellow brown loam soil, and the area of rocky desertification land accounts for 46.73% of the total area of rocky desertification land in the province, ranking first in the province [34]. The land type is mainly forest land, followed by farmland and grassland [35], and plants grown in the north and south are suitable for growth here. According to the differences in the planting habitats of vetiver grass, the sample plots are divided into the following categories: the original habitat (OH) retained after reservoir construction; the hydro-fluctuation belt (HB) affected by the construction of the reservoir itself; and the road slope (RS), which is used for the construction of reservoir-supporting facilities. Three replicates are performed for each habitat, totaling 9 sample points.

### 2.2. Sample Collection and Physicochemical Determination

Five sampling points are used for each sampling point. A 3 cm diameter soil drill is used to obtain 0–10 cm of surface soil, and the sampling point must be 5 cm away from the vetiver plant. Mix the 5 drilled soil samples evenly into one sample; remove debris, such as gravel and branches; divide them into two parts; put them into self-sealing bags; and bring them back to the laboratory. One is used for determining soil physicochemical properties, and the other is used for high-throughput sequencing of soil eukaryotic microbial amplicons. Each land use type had three replicates.

The laboratory uses the electrode potential method to determine soil pH, the potassium dichromate volumetric method to determine soil organic matter (SOM), the perchloric acid sulfuric acid digestion method to determine total nitrogen (TN) and total phosphorus (TP), the alkaline hydrolysis diffusion method to determine alkaline hydrolysis nitrogen (AN), 0.5 mol·L^−1^ NaHCO_3_ extraction and molybdenum antimony anti-chromogenic ultraviolet spectrophotometry to determine available phosphorus (AP), the NaOH melting atomic absorption method to determine total potassium (TK), and the ammonium acetate extraction method to determine available potassium (AK) The drying and weighing method is used to determine the soil water content (SWC) [36,37].

### 2.3. DNA Extraction and High-Throughput Sequencing

Each soil sample was weighed to obtain 0.5 g of fresh soil, and the total genomic DNA of the soil microbial community was extracted using the FastDNA^®^ SPIN Kit for Soil (MP Biomedicals, Santa Ana, CA, USA). PCR amplification of the 18S rRNA gene was performed using SSU0817F (5′-TTAGCATGGAATAATRRAATAGGA-3′) and SSU1196R (5′-TCTGGACCTGGTGAGTTTCC-3′) primers [38]. The following PCR reaction system is used: 2 μL 10× buffer, 2 μL 2.5 mmol·L^−1^ dNTPs, 0.8 μL upstream primer (5 μmol·L^−1^), 0.8 μL downstream primer (5 μmol·L^−1^), 0.2 μL rTaq polymerase, 0.2 μL BSA (Bovine Serum Albumin), 10 ng RTAq polymerase template DNA, and supplement with up to 20 μL ddH_2_O. The amplification procedure was as follows: 95 °C for 3 min, 37 cycles (95 °C 30 s, 55 °C 30 s, 72 °C 45 s), 72 °C for 10 min, and finally stored at 4 °C (PCR instrument: ABI GeneAmp^®^ 9700, Foster City, CA, USA).

Three replicates are set for each sample. After mixing the PCR products of the same sample, use 2% agarose gel to recover the PCR products. Use the AxyPrep DNA Gel Extraction Kit (Axygen Biosciences, Union City, CA, USA) to purify the recovered products. Use 2% agarose gel for electrophoresis detection. Use Quantum™ Fluorometer (Promega, Madison, WI, USA) to detect and quantify the recovered products. Illumina MiSeq sequencing technology from Shanghai Meiji Biopharmaceutical Technology Co., Ltd. (Shanghai, China) was used for high-throughput sequencing analysis (MiSeq PE300 sequencer).

### 2.4. Statistical Analysis

Univariate analysis of variance was used to examine the differences in chemical factors and eukaryotic microbial diversity indices under different habitats, while Tukey HSD was used for multiple comparisons between different sites. Kruskal–Wallis non-parametric test was used for data that still did not conform to the normal distribution after conversion, and Mann–Whitney tests were used to compare the differences between sample points. The above analysis was completed using IBM SPSS Statistics 23 (IBM, Armonk, NY, USA). Principal coordinate analysis (PCoA) of eukaryotic microbial communities based on Bray–Curtis distance between samples was performed using CANOCO 5.0. Redundancy analysis (RDA) is used to evaluate the impact of biochemical factors on the distribution of soil eukaryotic microbiota.

The vegan package [39] in R 3.6.3 calculates the species richness index (Chao1 index and Sobs index) and the diversity index (Shannon–Wiener index) of eukaryotic microorganisms, and the ANOSIM function is used to test whether the differences between groups are significant.

$$Shannon–Wiener\;index=-\sum_{i=1}^{Sobs}\frac{n_{i}}{N}ln\frac{n_{i}}{N}$$

$$Chao1\;index=Sobs+\frac{n_{1\left(n_{1}-1\right)}}{2\left(n_{2}+1\right)}$$

Here, Chao1 index is the number of OTUs (Operational Taxonomic Units) actually observed, *n*_1_ is the number of OTUs containing only one sequence, *n*_2_ is the number of OTUs containing only two sequences, *n_i_* is the number of sequences contained in the *i*th OTU, and *N* is the total number of sequences.

Mantel function calculates the correlation between eukaryotic microorganisms and chemical factors; ggplot2 package [40] draws correlation heat maps. Phych package [41] analyzed the co-occurrence patterns of eukaryotic microorganisms in different habitats, selected OTUs with relative abundance greater than 0.2%, and screened the correlation coefficient r > 0.6 and significance *p* < 0.05 to construct a co-occurrence network relationship of eukaryotic microorganisms at the boundary level based on Spearman correlation analysis. The igraph package calculates the network topology parameters of the network graph, including the modularity index, network density, average degree, and average clustering coefficient. Co-occurring networks were visualized using Gephi (Version 0.9.3).

## 3. Results

### 3.1. Soil Chemical Characteristics

There are significant differences in soil chemical factors among the different habitats, and there are significant differences in soil chemical factors except for TK among the three habitats (*p* < 0.05) (Table 1). The SOM, TN, AN, and AP values in the OH habitats are all the highest, whereas the pH, TP, and AK values are all the lowest. The pH, TP, AK, and SWC in the HB habitat increased compared to the OH habitat, and there was a significant difference between the two habitats. The pH, TP, and AK of the RS habitat increased, while the contents of SOM, TN, AN, AP, and SWC all decreased to the lowest levels. Except for SWC and TK, there were significant differences between the RS habitat and the OH habitat.

### 3.2. Quality Evaluation and Species Composition of Eukaryotic Microbial Sequencing

**Figure 1:**
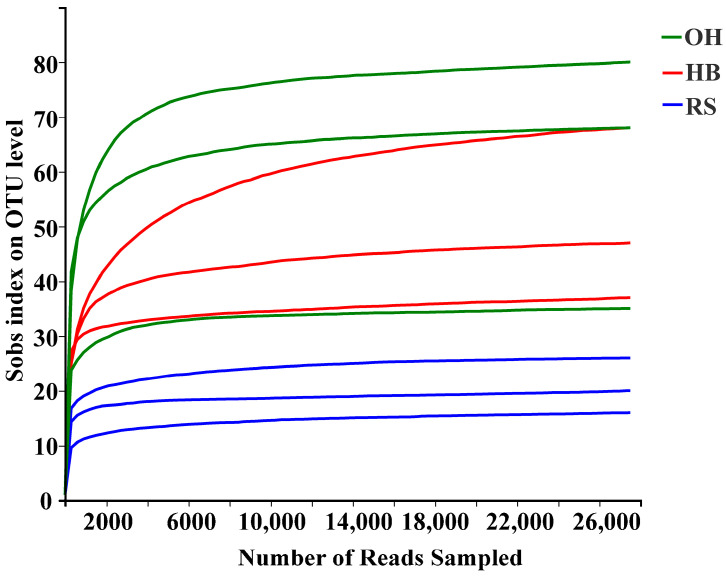
Dilution curves of Sobs at OTU levels for eukaryotic microbes in three habitats in the reservoir area.

A total of 325,526 optimized sequences were obtained from three different habitat soils, with a total base number of 130,706,139 and an average sequence length of 401 bp. The coverage of Good’s species is 99.97 ± 0.02%, and the Sobs index dilution curve of species richness tends to be flat (Figure 1). Both indicate that the detection rate of microbial communities in the environmental samples is close to saturation, and the current sequencing quantity can cover the vast majority of species in the sample.

**Figure 2:**
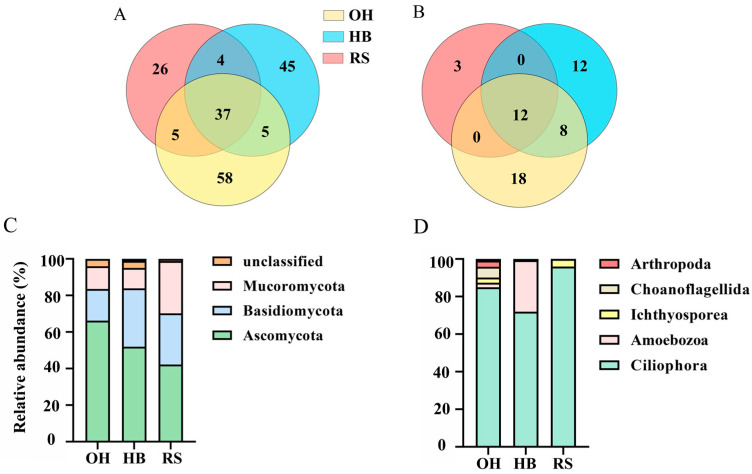
Community composition of fungi and soil microfauna in three habitats in the reservoir area. (**A**) Venn diagram of fungi OTUs; (**B**) Venn diagram of microfauna OTUs; (**C**) Relative abundance of fungal phyla; (**D**) Relative abundance of microfauna phyla.

A total of 139 different types of OTUs were identified through high-throughput sequencing, including 91 fungi, 29 soil microfauna, 5 microalgae, and 14 unclassified OTUs. Under the three habitat types, the number of fungal and soil microfauna OUT in the eukaryotic microbial community is significantly different. In terms of fungal composition, OH has the highest number of OTUs, followed by HB, and RS has the least (Figure 2A). Soil microfauna are similar to fungi, with the highest number of OTUs and the lowest number of RS in the OH (Figure 2B).

The five dominant phyla of eukaryotic microorganisms, Ascomycota, Basidiomycota, Mucormycota, Ciliophora, and Amoebozooa, together account for 96.31% of the total eukaryotic microbial community. The dominant phyla categories are consistent in all three habitats. The relative abundance of Ascomycetes among fungi in the OH habitat was the highest, reaching 66.24%, while the relative abundance decreased in the HB and RS. The relative abundance of Basidiomycota in the OH habitat is 17.30%, and both the HB and RS exhibit increased levels compared to it. Compared with OH, the relative abundance of Mucoromycota decreased in the HB, while the relative abundance was the highest in the RS (Figure 2C). The proportion of the three advantageous gates of the OH is as high as 95.93%, whereas values for the HB and OH are similar, with the proportion increasing to 98.83% in the RS. The relative abundance of Ciliophora in the soil microfauna of the OH habitat is 84.96%, while the relative abundance decreases in the HB. The relative abundance is the highest in the RS. The relative abundance of Amoebozoa in the OH and RS is relatively low, while the relative abundance is as high as 27.43% in the HB. The proportion of dominant phyla in the OH is 87.39%, with a maximum of 99.40% noted in the HB. Compared with the OH habitat, the value for the RS habitat also increased to 95.93% (Figure 2D).

### 3.3. Distribution and Diversity of Eukaryotic Microbial Communities

**Figure 3:**
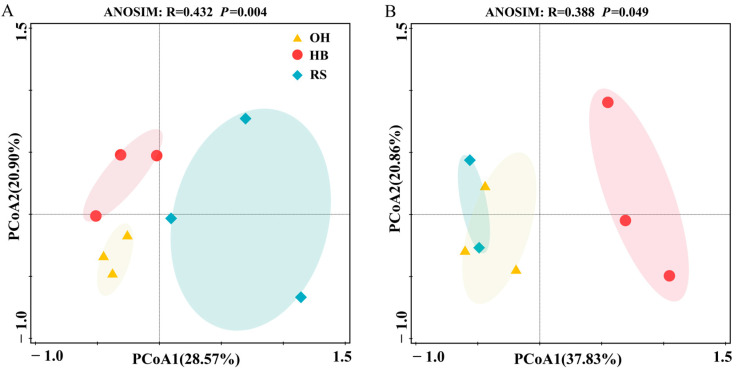
PCoA of eukaryotic microbial communities in three habitats in the reservoir area. PCoA was performed based on Bray–Curtis distances between samples, and ANOSIM was used to test the significant differences between groups. ▲, Original habitat; ●, Hydro-fluctuation belt; ◆, Road slope; (**A**) Fungi community; (**B**) Soil microfauna community.

The results of fungal community PCoA analysis (OTU level) showed that axis 1 explained a changing gradient of 28.57%, while axis 2 explained a changing gradient of 20.90%. There was significant differentiation in different habitats (Figure 3A) (ANOSIM, R = 0.432, *p* = 0.004). The fungal community composition in different habitats along axis 1 was clear, with OH located between HB and RS. The distribution of the three sampling points in the OH habitat is the most dense, and the differences in fungal communities between sampling points are the smallest. The distribution of the three sampling points in the HB and RS habitats is more dispersed than that in the OH habitat, and the differences in fungal communities between sampling points are significant.

The results of PCoA analysis (OTU level) of soil microfauna communities showed that axis 1 explained a 37.83% gradient of change, while axis 2 explained a 20.86% gradient of change. Different habitats also showed significant differentiation (Figure 3B) (ANOSIM, R = 0.388, *p* = 0.049), but the OH and RS habitat groups along axis 1 were not significant. The HB habitat is clearly distinguished from OH, and the distribution of sample points is more dispersed than the two. The differences in soil microfauna communities between HB sample points are the greatest.

The richness and diversity of fungi showed significant differences in different habitats (*p* < 0.05) (Table 2). The Sobs, Chao1, and Shannon–Wiener indices of fungi were the highest in the OH habitat. There was no significant difference between the HB and OH habitats, while the values for the RS habitats were significantly lower than OH. There was no significant difference in the Sobs and Chao1 indices of soil microfauna in different habitats, but the Shannon–Wiener index of RS was significantly lower than that of HB.

### 3.4. The Influence of Chemical Factors on the Structure of Eukaryotic Microbial Communities

**Figure 4:**
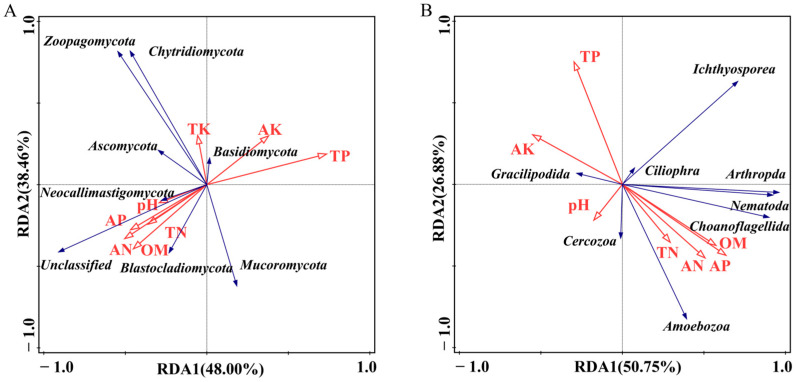
RDA biplot of eukaryotic microbial communities and chemical factors. (**A**) Fungi community; (**B**) Soil microfauna community.

Based on the abundance of different phyla of fungi and soil microfauna, removal trend correspondence analysis (DCA) was conducted, and the “Axis lengths” values were all less than 3. Therefore, RDA sorting was used to analyze the impact of chemical factors on fungi and soil microfauna. The RDA ranking of fungi shows that the explanatory power of Component 1 is 48.00%, and the explanatory power of Component 2 is 38.46% (Figure 4A). TK and SWC are positively correlated with Ascomycota, Basidiomycota, Zoopagomycota, and Chytridiomycota, while they are negatively correlated with Mucoromycota. SWC has the strongest positive correlation with Ascomycota, and TK has the strongest negative correlation with Mucoromycota. PH, AP, AN, and SOM are positively correlated with Neocollimastigomycota and Blastocladiomycota, while AK and TP are negatively correlated. pH has the strongest positive correlation with Neocollimastigomycota, whereas TP has the strongest negative correlation with it.

The RDA ranking of soil microfauna shows that the interpretability of Component 1 is 40.10%, and Component 2 is 23.80% (Figure 4B). AP, AN, SOM, and TN are positively correlated with Cercosoa, Ciliophora, Amoebozoa, Choanoflagellida, Arthropoda, Nematoda, and Ichthyosporota, while TP and AK are negatively correlated with Gracilipodida. SOM has the strongest positive correlation with *Actinophora*, while it has the strongest negative correlation with *Choanoflagellida*. There is a strong negative correlation between pH and SWC and *Ichthyosporea*.

**Figure 5:**
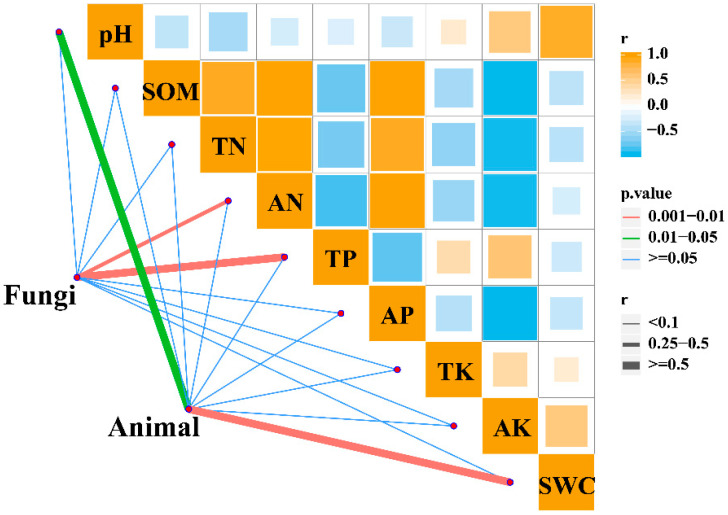
Mantel correlation of eukaryotic microbial communities with chemical factors.

Mantel analysis showed a significant correlation between eukaryotic microbial communities and chemical factors (R = 0.369, *p* = 0.01), as well as a significant correlation between chemical factors (R = 0.921, *p* = 0.001). There is a highly significant positive correlation between fungal communities and AN and TP. The soil microfauna community has a highly significant positive correlation with pH and SWC, and no significant correlation was noted with other chemical factors (Figure 5). There is a positive correlation between pH and SWC among chemical factors and a negative correlation between AN and TP.

**Figure 6:**
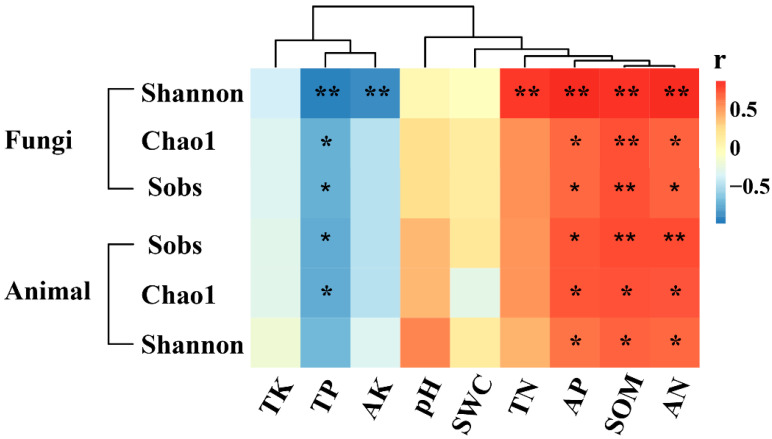
Spearman correlation heat map between diversity indices of soil fungi and microfauna and chemical factors. * *p* < 0.05; ** *p* < 0.01.

Spearman correlation analysis between the diversity index of eukaryotic microorganisms and chemical factors (Figure 6) found that AP, SOM, and AN were significantly positively correlated with the three diversity indices of eukaryotic microorganisms. TP is significantly negatively correlated with most diversity indices. AK only had a significant negative correlation with the Shannon–Wiener index of fungi, while TN only had a significant positive correlation with the Shannon–Wiener index of fungi.

### 3.5. Co-Occurrence Network of Eukaryotic Microorganisms

**Figure 7:**
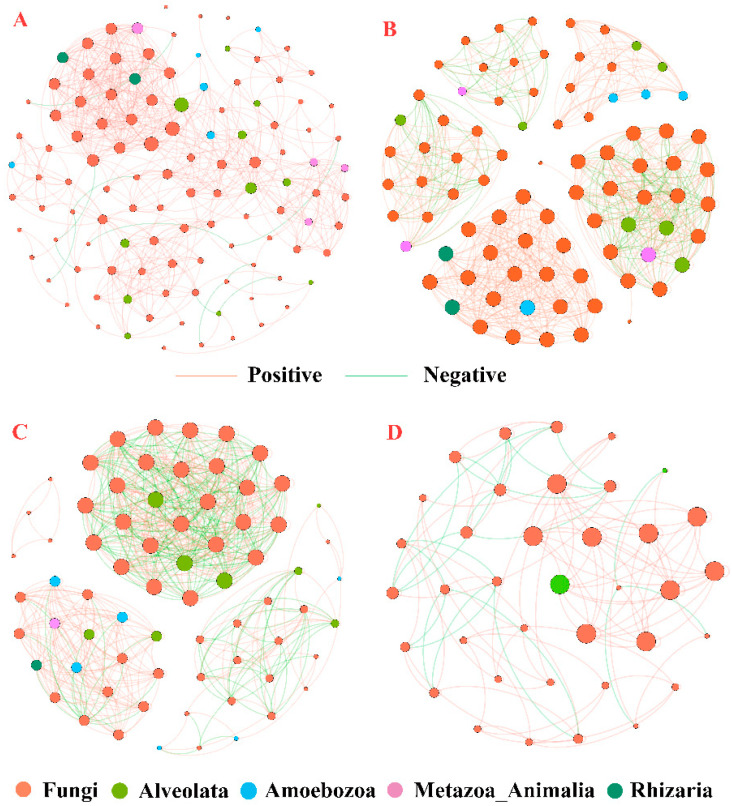
Co-occurrence networks based on the dominant kingdom of eukaryotic microbial OTUs. Network connections with Spearman correlation coefficient > |0.6| and *p* < 0.05 are shown. The red line represents a positive correlation, while the blue line represents a negative correlation. (**A**) Total habitat; (**B**) Original habitat; (**C**) Hydro-fluctuation belt; (**D**) Road slope protection.

The soil fungal and microfauna communities in all three habitats showed significant co-occurrence patterns, and compared with OH, the network relationships in HB and RS habitats showed significant changes (Figure 7). The OTU nodes in the co-occurrence network diagram belong to five kingdoms: Fungi, Alveolata, Amoebozooa, Metazoa_Animalia, and Rhizaria. In the comprehensive symbiotic network diagram of three habitats (Figure 7A), positive correlation connections between nodes dominate the network diagram. The number of node connections in OH habitats is higher than that in HB and RS habitats.

Further, the topological characteristics of the co-occurring network were calculated to evaluate the complexity of the soil eukaryotic microbial network (Table 3). Among the three habitats, the total occurrence network consists of 113 OTU nodes and 692 edges, of which 80.53% are fungal taxa and 19.47% are soil microfauna taxa. The modularity indices of the total habitat, OH, HB, and RS networks are all higher than the threshold of modularity indices (>0.4), indicating that the constructed symbiotic network has a modular structure. The association between different eukaryotic microbial communities in different habitats shown in the network diagram is significant and reliable. The network relationship between OH (Figure 7B) and HB (Figure 7C) habitats is the closest, and there is a complex and close relationship between the two eukaryotic microbial communities. The network density, average degree, and average clustering coefficient in the HB habitat are all the highest, reaching 0.278, 19.2, and 0.986, respectively, but the nodes and edges are slightly lower than those in the OH habitat. From OH to HB, the types of OTUs of Alveolata associated with fungi remained essentially unchanged, with a focus on Hypotrichia, Colpodea, and Prostomatea in Ciliophora, while the OTUs of Metazoa related to fungi were also largely preserved. However, the types of OTU in Rhizaria and Amoebozoa changed considerably, with fewer types of OTUs and the original related OTU groups disappearing (Figure S1B,C).

The network topology parameters of the RS habitat are lower than those of the OH habitat. The connections between eukaryotic microbial communities are looser than those of the OH habitat, but the proportion of positive correlation connections is higher than that of the OH habitat (Figure 7D). From OH to RS, the OTU types of microfauna associated with fungi declined dramatically, with only one type of OTU in Alveolata related to Hypotrichia being maintained (Figure S1B,D).

## 4. Discussion

In the soil ecosystem, fungi play a critical role in the breakdown of organic matter and the facilitation of nutrient cycling [9]. One common important metric used to assess changes in soil quality is the structural makeup of the fungus community [42,43]. Moreover, soil fungus actively contributes to the breakdown, modification, and transformation of organic matter, which is a critical component of the soil carbon and nutrient cycles [44]. However, research on the network relationship between soil fungi and microfauna communities in reservoirs planted with *Chrysopogon zizanioides* is limited. In the current study, following the construction of the reservoir, compared to the original habitat, the soil environment in the hydro fluctuation belt underwent significant modifications due to seasonal inundation. Compared to the original habitat, the soil pH, TP, and water content in the hydro-fluctuation belt increased, while the nitrogen content decreased. A previous study showed that nitrogen and phosphorus can affect the community structure of soil eukaryotic microorganisms by affecting their dominant phylum, thereby affecting biodiversity [45]. This study found that the distribution of fungal and soil microfauna communities was significantly different between the two habitats, and the abundance of the original habitat community was greater than that of the hydro-fluctuation belt. Fungi are the dominant group of eukaryotic microorganisms in the soil, and the dominant phyla of fungal communities in both habitats are consistent. However, the relative abundance of fungi in the deciduous zone has changed. The vast majority of fungi in this habitat belong to Ascomycota and Basidiomycota, which are closely related [46]. Treseder et al. [47] found that compared with other fungi, the spores of Ascomycota have thicker cell walls and are not significantly responsive to slight changes in the external environment, and they exist as the dominant bacteria in various types of soil. The dominant phyla of the two habitats were Ciliophora, and the relative abundance of Amoebozoa in the fluctuation zone was greater than that in the original habitat. Compared with fungi, soil microfauna were more sensitive to changes in soil pH and soil moisture. Similar to our study, Li et al. [48] also reported that soil moisture, organic matter, ammonia nitrogen, total nitrogen, and total phosphorus have significant impacts on the abundance of Ciliophora.

The complex relationship between eukaryotic microorganisms is an important feature of eukaryotic microbial communities [49], which can have a buffering ability against environmental interference. Changes in chemical parameters can affect the relationships between eukaryotic microorganisms, and changes in soil moisture content can affect nutrients and soil voids. As such, these modifications may have an impact on the direct interactions and network complexity of eukaryotic microbial communities [50,51]. The soil moisture in the hydro-fluctuation belt is significantly higher than that in the original habitat, and intermittent inundation may promote the rapid proliferation of soil fungi. In this study, it was found that the number of fungi in the hydro-fluctuation belt increased. Our results are similar to Liu et al. [52] who analyzed the changes in microbial communities in the Three Gorges Reservoir area and found that the total number of cultivable microorganisms decreased after flooding, but the number of fungi slightly increased. The interaction between eukaryotic microorganisms reflects the adaptability of eukaryotic microbial communities to environmental changes. Symbiotic network analysis has found that there are different interaction relationships and intensities between soil microfauna and fungi in the original habitat and the habitat of the hydro-fluctuation belt, which will also affect the function and stability of soil ecosystems. In co-occurring networks, positively correlated interactions are mainly seen as cooperation, while negatively correlated interactions are seen as competition [53]. The eukaryotic microbial communities’ interspecific interactions in the original habitat and the hydro-fluctuation belt show favorable correlations that mostly point to co-aggregation, cross-feeding, companion migration, and co-evolution among these species [54,55].

The construction of infrastructure to sustain reservoirs will alter the soil’s chemical composition, which will alter the community’s eukaryotic microorganism composition [56]. In addition to organic matter, there is also a rich presence of soil phosphorus in the soil mineral layer. Therefore, our findings show that the use of road slope protection increases the amount of mineral phosphorus that is exposed to the soil, increasing the total amount of phosphorus. Both phosphorus and nitrogen can have an impact on the variety and spread of fungal communities in the soil. Similar to our results, Dang et al. [57] also found a significant correlation between soil organic carbon, TN, and AP and soil fungal communities when studying artificial forest soils, with soil AP content being the key factor leading to differences in soil fungal communities. In addition, Liu et al. [58] found that a soil available phosphorus content greater than 53.6 mg·kg^−1^ was the threshold for significant changes in fungal diversity when studying typical paddy soil microorganisms. The current study found that the effective phosphorus content of road slope protection was much lower than that of the original habitat, as well as below the study’s threshold. Although the predominant fungal species under road slope protection are consistent with the original environment, the number of fungal groups and diversity index is much lower than in the original habitat. There is a complex interrelationship between soil microfauna and their living environment, and the *Ciliophora* is one of the three major groups of soil protozoa, with huge biomass and productivity. Zhang et al. [59] found that soil organic matter, total nitrogen, and total phosphorus contents are positively correlated with the type, quantity, and biomass of soil microfauna. Additionally, this study showed that the road slope protection had the lowest amounts of total nitrogen content and soil organic matter. Furthermore, the number of groups in the soil microfauna community was significantly decreased, and it was restricted to just two phyla: *Ciliophora* and *Ichthyosporea*. However, compared with the original habitat, no significant differences in diversity were found. This may be due to the preference of *Ciliophora* in arid and semi-arid soil environments. Thus, the drier soil environment of the RS habitat caused a significant decrease in the number of microfauna but had little effect on *Ciliophora* [60]. Most *Ichthyosporea* are animal parasites that can be parasitic to aquatic and land animals; hence, their presence in soil is primarily dependent on the presence of living animals nearby, with minimal relevance to the physical and chemical qualities of the soil [61].

After the construction of supporting facilities for the reservoir, the water content and organic matter content of the road slope protection habitat were reduced to the lowest levels, which affects the network relationship between eukaryotic microorganisms. Brown et al. [60] believe that when soil organic matter content is low, the competition between communities in such environments is weak, and strong mutual cooperation can enable eukaryotic microbial communities to better resist changes in the external environment and maintain the stability of microbial networks [26].

In the road slope protection habitat, there are many positive correlations between eukaryotic microorganisms, which may be attributed to cooperation. However, the average degree and clustering coefficient of the eukaryotic microbial network for the road slope protection habitat, as well as the nodes and related edges, showed considerable declines in comparison to their native habitat. Similarly, the network’s complexity was dramatically reduced, resulting in thinner ecological links. Furthermore, the network is dominated by interactions between fungi, with only a few linkages between animal taxa. Han et al. [62] reported the relationship between soil animal community characteristics and 13 environmental factors in low mountainous areas of the Changbai Mountains, and different environmental factors have a significant impact on soil animals. Luo et al. [20] also found that the individual and group numbers of soil animals in the Nanniwan wetland are closely related to the degree of wetland damage. As the degree of wetland damage increases, the number of soil animal groups and individuals gradually decreases, and the stability of the ecosystem decreases. The above research reflects that the decreased network connectivity after the deployment of road slope protection is partly connected with a reduction in soil animal populations. As a result, the stability of the ecosystem is impacted by the decline in soil animals, which also leads to a decline in the complexity of the soil food web.

## 5. Conclusions

In summary, the study shows that the construction and operation of reservoirs have affected chemical factors, such as nitrogen, phosphorus, organic matter, pH, and water content, in the soil, thereby altering the community composition and diversity of soil fungi and soil microfauna. After the construction of the reservoir, the newly formed hydro-fluctuation belt has reduced the types of interrelationships between fungi and animals compared to the original habitat and is more concentrated in the interrelationships between the fungal kingdom and the vesicular algae kingdom. Although vegetation is also planted in the road slope protection area of the reservoir construction supporting project, due to the damage to and loss of the organic layer, the eukaryotic microbial community has significantly decreased, with the most significant decrease noted in soil microfauna, resulting in a simple structure of the soil food web and affecting the function and stability of the soil ecosystem.

## Figures and Tables

**Table 1:** Soil chemical characteristics in three habitats in the reservoir area.

Chemical Factor	OH	HB	RS
pH	8.20 ± 0.08 b	8.61 ± 0.08 a	8.23 ± 0.02 a
SOM/g·kg^−1^	132.77 ± 9.14 a	59.50 ± 8.34 b	36.26 ± 4.39 b
TN/g·kg^−1^	2.03 ± 0.16 a	1.01 ± 0.00 b	0.83 ± 0.04 b
AN/mg·kg^−1^	139.77 ± 2.03 a	86.57 ± 4.87 b	56.47 ± 2.07 c
TP/g·kg^−1^	0.97 ± 0.03 c	1.28 ± 0.08 b	2.56 ± 0.03 a
AP/mg·kg^−1^	75.10 ± 4.91 a	41.64 ± 1.03 a	28.93 ± 0.87 b
TK/g·kg^−1^	7.03 ± 0.30	14.00 ± 3.50	16.50 ± 5.73
AK/mg·kg^−1^	57.41 ± 2.41 b	96.62 ± 1.84 a	97.67 ± 2.53 a
SWC/%	8.07 ± 0.62 b	15.78 ± 0.22 a	7.30 ± 0.27 b

Note: Different lowercase letters in the same row indicate a significant difference (*p* < 0.05), *n* = 3. Data in the table are mean ± standard error.

**Table 2:** Diversity indices of soil eukaryotic microbes in three habitats in the reservoir area.

Eukaryotes	Diversity Index	OH	HB	RS
Fungus	Sobs	44.67 ± 8.37 a	39.33 ± 5.24 ab	19.33 ± 2.40 b
	Chao1	45.00 ± 8.66 a	40.50 ± 4.44 ab	20.00 ± 2.08 b
	Shannon–Wiener	2.88 ± 0.09 a	2.37 ± 0.16 ab	1.88 ± 0.08 b
Soil microfauna	Sobs	9.33 ± 3.28	8.33 ± 2.40	1.33 ± 0.67
	Chao1	9.67 ± 3.38	10.00 ± 4.04	1.33 ± 0.67
	Shannon–Wiener	1.20 ± 0.29 ab	1.42 ± 0.18 a	0.39 ± 0.20 b

Note: Different lowercase letters in the same row indicate a significant difference (*p <* 0.05), *n* = 3. Data in the table are mean ± standard error.

**Table 3:** Topological characteristics of eukaryotic microbial network diagrams in three habitats in the reservoir area.

Habitat Type	Node	Edge	Modularization Index	Network Density	Average Degree	Average Clustering Coefficient	Positive Correlation Connection	Negative Correlation Connection
Total habitat	113	692	0.619	0.109	12.248	0.775	682 (98.41%)	10 (1.59%)
Original habitat	84	773	0.944	0.222	18.405	0.984	611 (79.04%)	162 (20.96%)
Hydro-fluctuation belt	70	672	1.703	0.278	19.200	0.986	437 (65.03%)	235 (34.97%)
Road slope protection	37	144	0.772	0.216	7.784	0.953	122 (84.72%)	22 (15.28%)

## Data Availability

Data are contained within the article and Supplementary Materials.

## Supplementary Materials

The following supporting information can be downloaded at: https://www.mdpi.com/article/10.3390/plants13030393/s1, Figure S1: Co-occurrence networks of based on the dominant kingdom of eukaryotic microbial OTU.
