# Supplementary figures and images for: Network of Soil Fungi and the Microfauna Community under Diverse Anthropic Disturbances under Chrysopogon zizanioides Planting in the Reservoir
Source: Plants (Basel). 2024 Jan 29;13(3):393. doi: 10.3390/plants13030393 (PMC10857095; doi:10.3390/plants13030393)

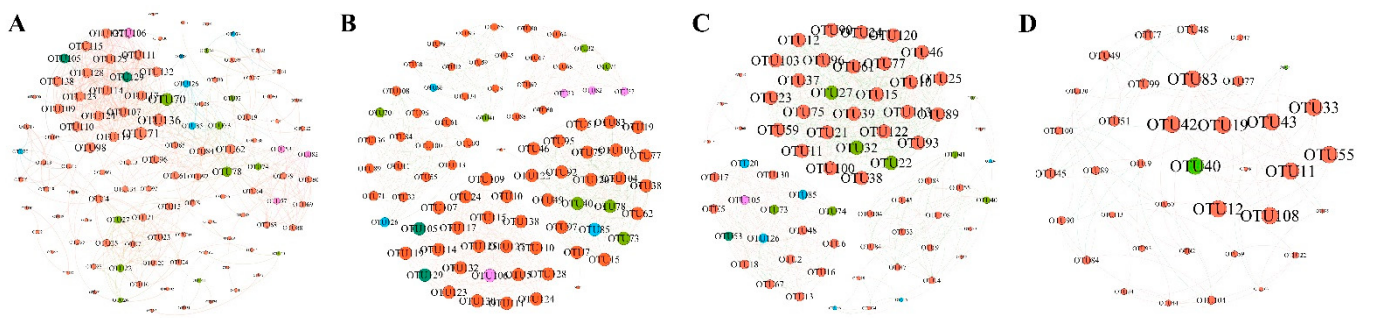

**Figure S1 Co-occurrence networks of based on the dominant kingdom of eukaryotic microbial OTU.**

Supplement: Supplementary file 1 [file plants-13-00393-s001.zip › plants-2800285-supplementary.pdf]
